# Low-Intensity Peer-Led Exercise Improves Fall Risk Appraisal in Older Women

**DOI:** 10.1093/geroni/igaf122.3807

**Published:** 2025-12-31

**Authors:** Chitra Banarjee, Kworweinski Lafontant, Jethro Raphael Suarez, Rui Xie, Ladda Thiamwong

**Affiliations:** University of Central Florida, Orlando, Florida, United States; University of Central Florida, Orlando, Florida, United States; University of Central Florida, Florida, United States; University of Central Florida, Orlando, Florida, United States; University of Central Florida, Orlando, Florida, United States

## Abstract

About 64-79% of older adults aged 60+ have maladaptive fall risk appraisal (FRA), a discrepancy of perceived and physiological fall risks, leading to reduced physical activity and multiple falls. Older adults have been classified into four groups of FRA by integrating fear of falling (FoF) and balance, as measured by the Short Falls Efficacy Scale-International, and BTrackS Balance Test (BBT); a) rational (low FoF and normal balance), b) incongruent (low FoF despite poor balance), c) irrational (high FoF despite normal balance), and d) congruent (high FoF and poor balance). This study examined the effect of an 8-week low-intensity peer-led intervention (PEER) on changes in FRA. We recruited 314 community-dwelling older women (mean age=74.3±6.9years) at four timepoints (baseline and follow-ups at 2, 4, and 6 months) to evaluate longitudinal changes in quantified FRA, or FRA distance, a quantified FRA adapted using the distance formula, where greater values indicate composite deficits in FoF and balance. A linear mixed effects model included fixed effects of intervention, original FRA group, age, and race, with random effects of participant and timepoint. The intervention shows a slight but significant reduction in FRA distance (β=-2.59, p = 0.047), indicating improved outcomes. Compared to the rational group, all other original FRA groups—except irrational—had significantly worse FRA scores (p < 0.001), and advanced age was associated with worse FRA distance (p < 0.001). These results demonstrate the potential of the PEER intervention for shifting FRA, but the limited size of the effect suggests that greater exercise intensity may further improve FRA outcomes.

